# Valorization of *Gelidium amansii* for dual production of D-galactonic acid and 5-hydroxymethyl-2-furancarboxylic acid by chemo-biological approach

**DOI:** 10.1186/s12934-020-01357-6

**Published:** 2020-05-14

**Authors:** Peng Liu, Jiaxiao Xie, Huanghong Tan, Feng Zhou, Lihua Zou, Jia Ouyang

**Affiliations:** 1 College of Forestry, Nanjing Forestry University, Nanjing, 210037 People’s Republic of China; 2 Key Laboratory of Forestry Genetics & Biotechnology (Nanjing Forestry University), Ministry of Education, Nanjing, 210037 People’s Republic of China; 3 Jiangsu Co-Innovation Center of Efficient Processing and Utilization of Forest Resources, College of Chemical Engineering, Nanjing Forestry University, Nanjing, 210037 People’s Republic of China; 4 Jiangsu Province Key Laboratory of Green Biomass-based Fuels and Chemicals, Nanjing, 210037 People’s Republic of China

**Keywords:** *Gelidium amansii*, D-galactonic acid, 5-hydroxymethyl-2-furancarboxylic acid, *Pseudomonas putida*, Biotransformation

## Abstract

**Background:**

Marine macroalgae *Gelidium amansii* is a promising feedstock for production of sustainable biochemicals to replace petroleum and edible biomass. Different from terrestrial lignocellulosic biomass, *G. amansii* is comprised of high carbohydrate content and has no lignin. In previous studies, *G. amansii* biomass has been exploited to obtain fermentable sugars along with suppressing 5-hydroxymethylfurfural (HMF) formation for bioethanol production. In this study, a different strategy was addressed and verified for dual production of D-galactose and HMF, which were subsequently oxidized to D-galactonic acid and 5-hydroxymethyl-2-furancarboxylic acid (HMFCA) respectively via *Pseudomonas putida.*

**Results:**

*G. amansii* biomass was hydrolyzed by dilute acid to form D-galactose and HMF. The best result was attained after pretreatment with 2% (w/w) HCl at 120 °C for 40 min. Five different *Pseudomonas* sp. strains including *P. putida* ATCC 47054, *P. fragi* ATCC 4973, *P. stutzeri* CICC 10402, *P. rhodesiae* CICC 21960, and *P. aeruginosa* CGMCC 1.10712, were screened for highly selective oxidation of D-galactose and HMF. Among them, *P. putida* ATCC 47054 was the outstanding suitable biocatalyst converting D-galactose and HMF to the corresponding acids without reduced or over-oxidized products. It was plausible that the pyrroloquinoline quinone-dependent glucose dehydrogenase and undiscovered molybdate-dependent enzyme(s) in *P. putida* ATCC 47054 individually played pivotal role for d-galactose and HMF oxidation. Taking advantage of its excellent efficiency and high selectivity, a maximum of 55.30 g/L d-galactonic acid and 11.09 g/L HMFCA were obtained with yields of 91.1% and 98.7% using *G. amansii* hydrolysates as substrate.

**Conclusions:**

Valorization of *G. amansii* biomass for dual production of D-galactonic acid and HMFCA can enrich the product varieties and improve the economic benefits. This study also demonstrates the perspective of making full use of marine feedstocks to produce other value-added products.

## Background

Production of biofuels and biochemicals from inexpensive biomass has attracted considerable attention to achieve sustainable economic growth and development in the world. In view of the inherent problems associated with terrestrial feedstocks, attention has been turned to marine macroalgae (seaweeds) as an alternate source of non-lignocellulosic feedstock for production of bioproducts [1, 2]. Compared with terrestrial feedstocks, macroalgae has superior characteristics as advanced feedstocks, including no lignin, high carbohydrate content, rapid grown rate with fixation of more CO_2_ than wood-biomass, and easy cultivation without the need of arable land and freshwater [3, 4].

*Gelidium amansii* and *Gracilaria gracilis* are two well-known agarophyte red macroalgae, which comprise polysaccharide complexes of fibrin (cellulose) and agar (agarose). Agar, the main component of agarophyte red macroalgae, is composed of D-galactose and 3,6-anhydro-l-galactose (AHG) with alternate α-1,3- and β-1,4-linkages [5]. D-galactose is fermentable mono sugar that can be converted to bioethanol and value-added chemicals, while AHG cannot be fermented by commonly fermentative microorganisms such as *Escherichia coli* and *Saccharomyces cerevisiae*. However, AHG is acid-labile and tends to be decomposed into 5-hydroxymethylfurfural (HMF) and, subsequently, into organic acids such as levulinic acid and formic acid [6]. Therefore, many studies focused on suppressing the inhibitor generation during pretreatment of red macroalgae and investigating the effects of HMF on fermentative process [7].

Different from the role of fermentation inhibitor, HMF is also one of the most important bio-based platform compounds. Due to the presence of two active groups on furan ring, hydroxymethyl and aldehyde, HMF can be oxidized to various furan intermediates, such as 2,5-diformylfuran, 5-hydroxymethyl-2-furancarboxylic acid (HMFCA), 5-formyl-2-furancarboxylic acid, and 2,5-furandicarboxylic acid (FDCA) [8]. Among them, HMFCA, formed by selective oxidation of the aldehyde group on HMF, is a less known oxidative product, which has potential applications in fields of material and pharmaceuticals [8]. Similarly, in addition to conventionally fermented into ethanol, D-galactose can be selectively oxidized to D-galactonic acid, which is chemically similar to D-gluconic acid, and thereby has the potential to be used in a variety of similar applications, such as in food, pharmaceutical, detergent, textile, leather, and concrete industries [9].

**Fig. 1:**
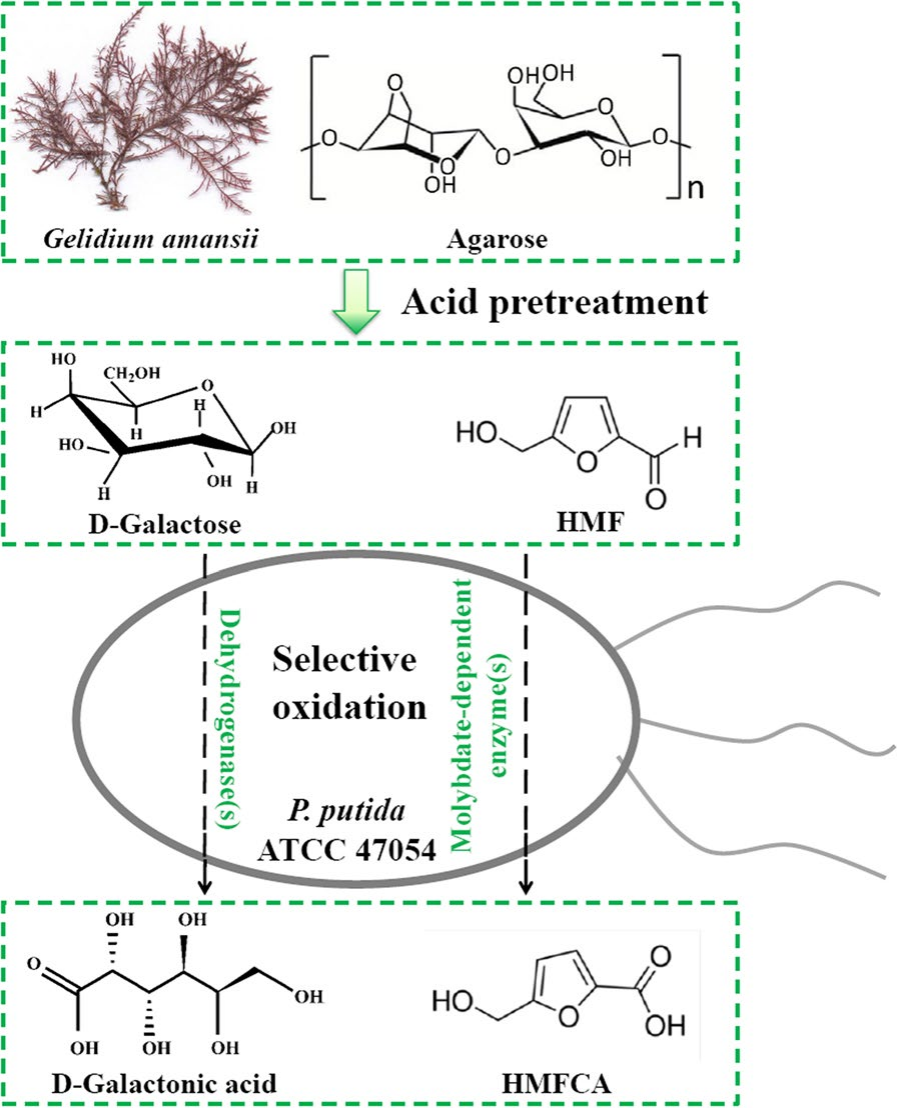
The schematic diagram of co-production of D-galactonic acid and HMFCA from *Gelidium amansii* biomass

Co-production of multi-products contributes to the competiveness of chemical engineering or bioengineering processes, which has been widely studied in upgrading of low-grade biomass [10–12]. Considering D-galactonic acid is the oxidative product of D-galactose while HMFCA is the oxidative product of HMF, herein, we reported an approach for dual production of D-galactonic acid and HMFCA from *G. amansii* by a chemo-biological method (Fig. 1). *G. amansii* was acid-hydrolyzed to form a mixture of D-galactose and HMF, which were subsequently converted into D-galactonic acid and HMFCA individually using *Pseudomonas* sp. Five species of *Pseudomonas* were screened for their ability to catalyze the oxidation of both D-galactose and HMF. The most promising candidate *P. putida* ATCC 47054 was used for the oxidation of *G. amansii* hydrolysates and the enzymes in *P. putida* ATCC 47054 involved in D-galactose and HMF selective oxidation was preliminarily investigated for the first time. The reaction was also evaluated at increased hydrolysates concentration.

## Results and discussion

### Pretreatment of *G. amansii* to produce D-galactose and HMF

**Fig. 2:**
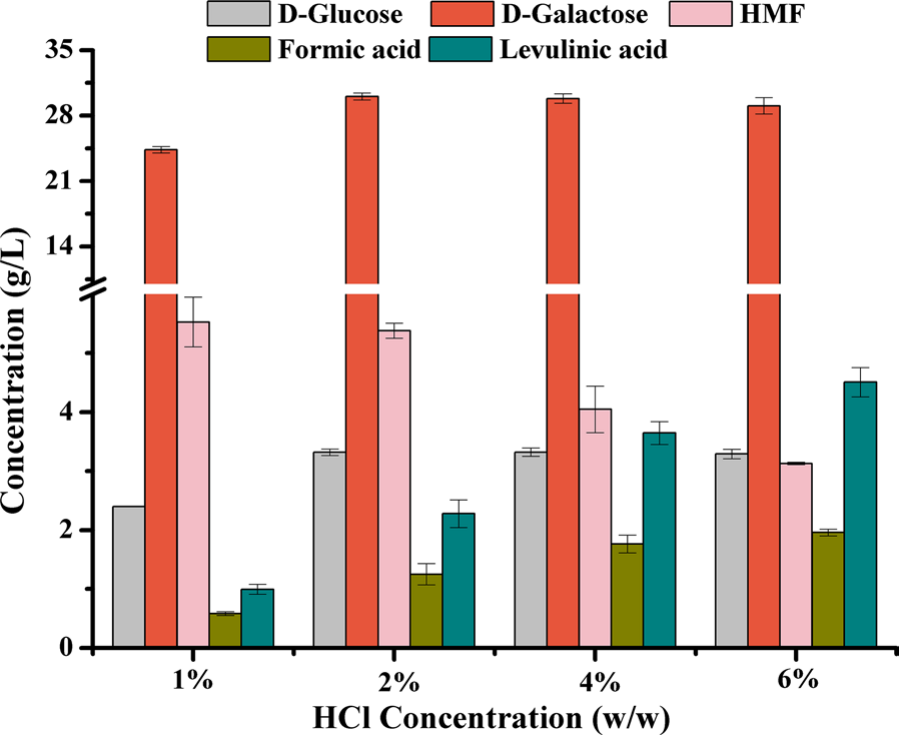
Results of dilute HCl pretreatment of *G. amansii*. Reaction conditions: 2 g of *G. amansii* in 30 mL of dilute HCl with different concentrations (1%, 2%, 4%, 6% w/w) at 120 °C for 1 h. Error bars represented standard deviations

The feasibility of D-galactose and HMF co-production was tested with four different HCl concentrations (1%, 2%, 4%, 6% w/w) at 120 °C for 1 h. Figure 2 revealed that improving the dosage of HCl from 1 to 2% (w/w) increased the concentration of D-galactose by 23.4%, from 24.35 g/L to 30.04 g/L, and further improvement of HCl dosage almost had no impact on D-galactose yield. Conversely, the HMF concentration decreased from 5.53 to 3.13 g/L with the concomitant increase in the concentrations of levulinic acid and formic acid, suggesting a degradation process of HMF, especially improving the dosage of HCl from 2% (w/w) to much higher. Beyond that, d-glucose generated from fiber was detectable, approximately 2.40 to 3.32 g/L. The improved HCl concentration resulted in obvious levulinic acid and formic acid formation, but no other by-products could be observed, which also indicated that the physical structure of *G. amansii* was almost absence of lignin.

**Fig. 3:**
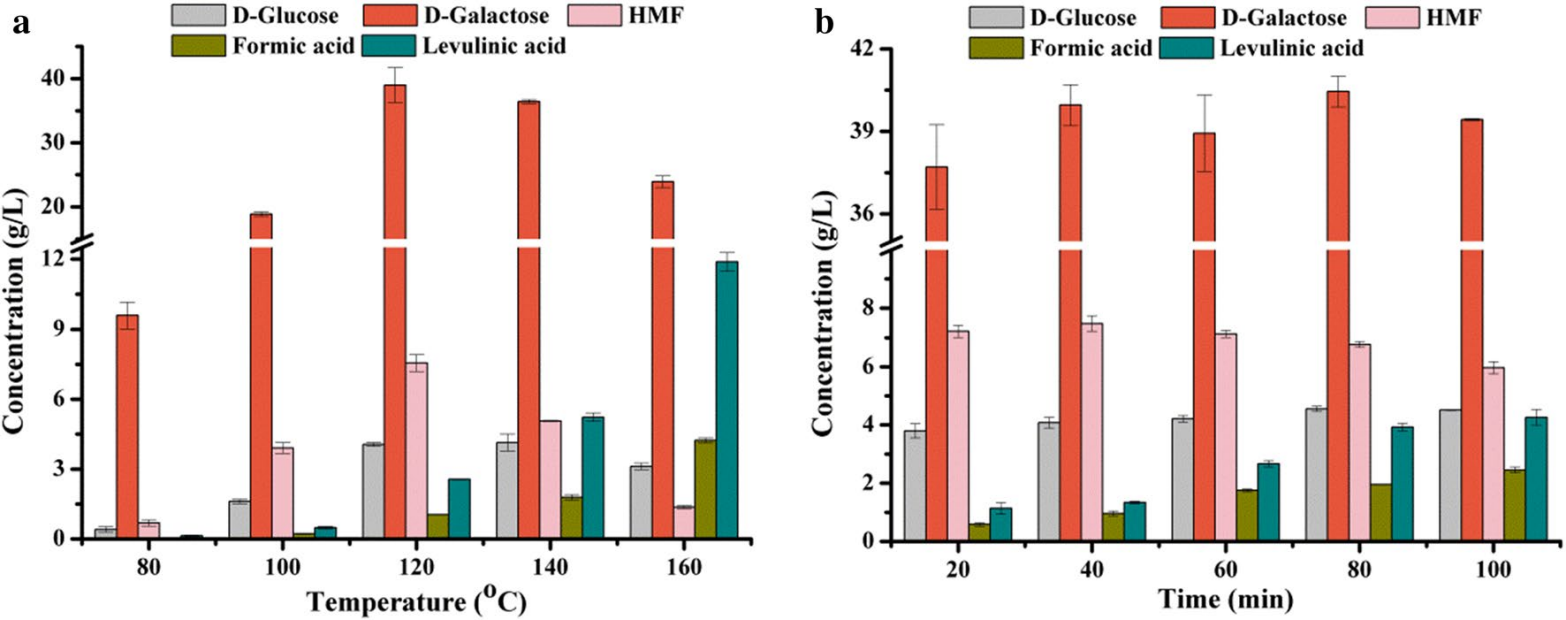
Effect of temperature and time on dilute HCl pretreatment of *G. amansii*. Reaction conditions: 2 g of *G. amansii* in 20 mL of dilute HCl with concentration of 2% (w/w) at different temperature for 1 h (**a**); 2 g of *G. amansii* in 20 mL of dilute HCl with concentration of 2% (w/w) at 120 °C for different time (**b**). Error bars represented standard deviations

With the aim of achieving both higher yield of D-galactose and HMF, effects of temperature and time on hydrolysis of *G. amansii* were evaluated. Figure 3a exhibited improving the temperature from 80 °C to 160 °C gave similar trend on D-galactose and HMF production. Both the highest concentration of D-galactose (38.99 g/L) and HMF (7.56 g/L) were obtained at 120 °C. Temperature lower than 100 °C was too facile for degradation of galactan and conversion of AHG in *G. amansii*. In contrast, when temperature was higher than 140 °C, a gradual decrease of D-galactose and HMF was observed, suggesting a further degradation process of D-galactose to undetected compounds, and a transformation of HMF to levulinic acid and formic acid.

Compared with temperature, effect of pretreatment time on *G. amansii* hydrolysis was insignificant (Fig. 3b). The holding time prolonged from 20 min to 40 min brought about slight enhancement of D-galactose and HMF concentration, and further extension of time had no influence on D-galactose and negative impact on HMF production, respectively. Therefore, a relatively high titer of 39.96 g/L D-galactose and 7.48 g/L HMF, with individual yields of 82.87% and 40.71%, was achieved when holding time was 40 min.

Based on the obtained experimental results, it can be observed that the concentration of levulinic acid was much improved under higher temperatures than other conditions, from 2.56 g/L (120 °C) to 11.88 g/L (160 °C) (Fig. 3a). It should be noted that the increased production of levulinic acid could not be compensated from HMF decomposition (7.56 g/L at 120 °C *vs* 1.37 g/L at 160 °C). It was possible that the combination of HCl and high temperature (160 °C or higher) can drive a further dehydration of the formed D-galactose intermediate (38.99 g/L at 120 °C vs 23.93 g/L at 160 °C) during the pretreatment process to generate HMF and subsequently levulinic acid. These results were in agreement with previous studies pointing to red algae as a potential biomass for levulinic acid production, which is another versatile platform chemical with numerous application fields [4].

### Oxidation of D-galactose and HMF using *Pseudomonas* sp. strains

In terms of oxidizing D-galactose and HMF into D-galactonic acid and HMFCA, it is desirable to explore strains that possess strong and specific activity towards the aldehyde group. As typically aerobic bacteria, the genus *Pseudomonas* can oxidize a wide range of aldose sugars into their corresponding aldonic acids [13, 14], but their potential capacities of HMF oxidation have not been fully investigated. In this study, five different species from *Pseudomonas* sp. were screened as putative candidates for both D-galactose and HMF oxidation. Additional file 1: Table S1 revealed that the D-galactose oxidation ability was universal in all tested strains. In contrast, only *P. putida* ATCC 47054 and *P. rhodesiae* CICC 21960 were capable of converting HMF into HMFCA, and the former had a little advantage. On the basis of the above results, *P. putida* ATCC 47054 was chosen as the biocatalyst for further investigations. Over-oxidation or further degradation reaction(s), which were readily caused by the complex enzyme systems in the cells, didn’t occur in the whole-cell biotransformation of *P. putida* ATCC 47054, which might be ascribed to the paucity of enzymes for assimilation of D-galactonic acid and HMFCA. Certain strains, such as *P. saccharophila*, *Azotobacter vinelandii*, *Caulobacter crescentus* and *E. coli*, possess a De Ley-Doudoroff route for dehydration of D-galactonic acid to produce 2-keto-3-deoxygalactonic acid, and the latter was further phosphorylated and cleaved to form glyceraldehyde-3-phosphate and pyruvate [15–18]. These strains can utilize D-galactose or D-galactonic acid as carbon source for growth. Nevertheless, this is not the case for *P. putida* ATCC 47054, which is devoid of enzymes involved in De Ley-Doudoroff pathway [19]. In the case of HMFCA, it can be converted to FDCA by full oxidation of the hydroxymethyl group on furan ring by *Cupriavidus basilensis* HMF14 and *Raoultella ornithinolytica* BF60, but the strains with such characteristic is rather rare [20, 21]. As a matter of fact, researchers have devoted many efforts to screen such strains or related enzymes for FDCA biosynthesis [20, 22–24]. For *P. putida* ATCC 47054, only the oxidation of aldehyde group on furan ring can be accomplished, which is similar to previously reported *Comamonas testosterone* SC1588 and *Gluconobacter oxydans* DSM 50049 [25, 26].

**Fig. 4:**
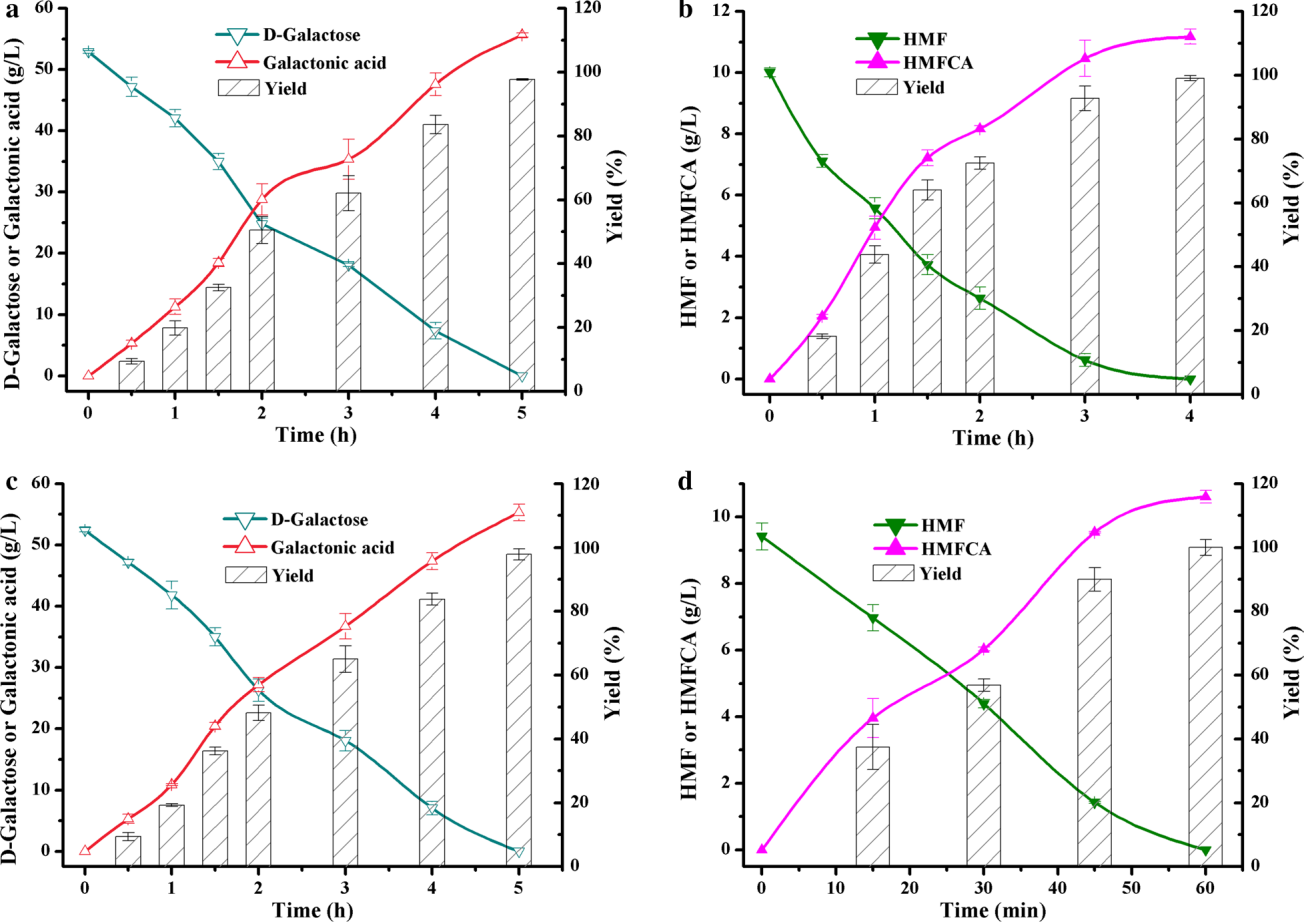
Profiles of selective oxidation of D-galactose to D-galactonic acid and HMF to HMFCA using *P. putida* ATCC 47054 cells without (**a**, **b**) or with HMF induction (**c**, **d**). Reaction conditions: 5 mL of phosphate buffer (200 mM, pH 6.0) containing 8 g_cdw_/L cells, 50 g/L D-galactose, 10 g/L HMF, and CaCO_3_ at half molar concentration of substrate, 35 °C and 200 rpm. Error bars represented standard deviations

Co-oxidation of D-galactose and HMF was conducted by *P. putida* ATCC 47054. The concentration of D-galactose and HMF in reaction mixtures was set according to their ratio in pretreated *G. amansii* hydrolysates. Figure 4a, b showed the co-production process of D-galactonic acid and HMFCA. It was found that 50 g/L D-galactose was transformed completely in 5 h, while 10 g/L HMF was converted totally in 4 h, with yields of 97.7% and 99.1%, respectively. Zhang et al. reported that the catalytic activities of *C. testosterone* SC1588 towards HMF were enhanced markedly when they were cultivated with a low concentration of furfural and furfural alcohol as the inducers [25]. Herein, we attempted to facilitate the oxidation reactions by supplementing 4.5 mM HMF, hoping to induce the expression of the enzyme(s) responsible for the aldehyde group oxidation. Indeed, the bioconversion ability towards HMF was improved significantly when HMF was added during the cultivation period, but no positive effect was detected for D-galactose bioconversion (Fig. 4c, d). Figure 4d showed that the reaction period of HMF bioconversion decreased from 4 h to 1 h with the induced cells as biocatalyst. It was plausible that enzyme(s) responsible for oxidation the aldehyde group of HMF and D-galactose were different. To sum up, no matter the cells were induced or not, the substrates were consumed completely, and the maximal D-galactonic acid and HMFCA yield were above 97% in all cases.

Although good yields were obtained for both D-galactonic acid and HMFCA, the main enzymes in *P. putida* ATCC 47054 involved in D-galactose and HMF oxidation are still unknown. In our another research that focused on the role of molybdate transport system in biotransformation of furanic aldehydes, we found that deletion of *modABC* (encoding a molybdate ABC transporter, PP_3828–PP_3830) could prevent the oxidation of HMF to HMFCA (unpublished data, manuscript in preparation) whereas no impact on D-galactose oxidation occurred. It is likely that one or more unknown molybdate-dependent oxidoreductases can specifically accept HMF as substrate. But frustratingly, we had not identified the target enzyme(s) responsible for HMF oxidation at present. In the case of D-galactose, in literatures, the oxidoreductases from bacteria that can oxidize the C1-position of D-galactose are D-galactose dehydrogenase and L-arabinose dehydrogenase [16, 27–29], but none of them can be searched on the genome of *P. putida* ATCC 47054 (GenBank no. AE015451.2). We then turned our attention to the pyrroloquinoline quinone-dependent glucose dehydrogenase (PQQ-GCD), which can transform a diverse of aldoses into their corresponding aldonic acids [13, 30]. A *gcd*-disrupted mutant was constructed by homologous recombination to investigate the role of PQQ-GCD in d-glucose oxidation. The oxidative activities of *P. putida* ATCC 47054 and the *gcd*-disrupted mutant towards D-galactose were determined, respectively. Compared with the wild strain that possessed the activity of 0.13 U/mg, the *gcd*-disrupted mutant appeared to have lost the ability to oxidize D-galactose in a large extent, evidenced by the low activity of 8.9 × 10^−4^ U/mg, which verified the pivotal role PQQ-GCD in D-galactonic acid production.

### Dual production of D-galactonic acid and HMFCA from *G. amansii* hydrolysates

**Fig. 5:**
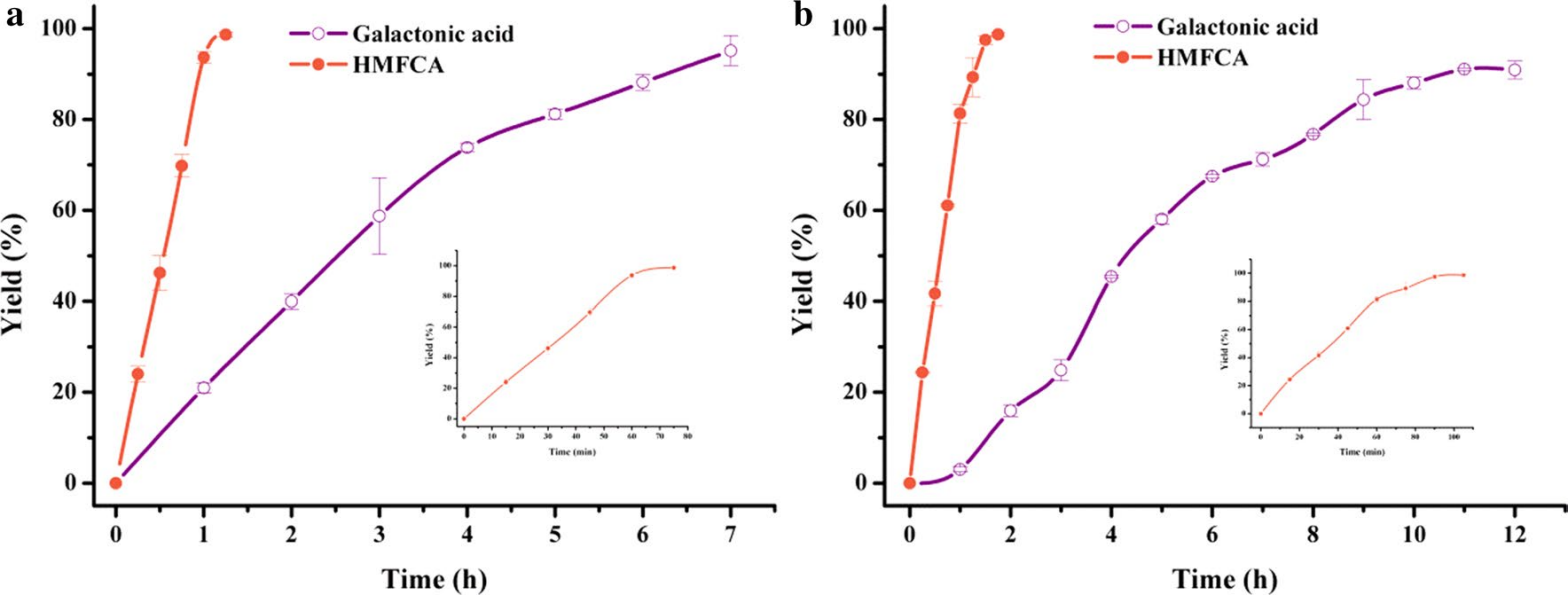
Co-production of D-galactonic acid and HMFCA from *G. amansii*, which was pretreated at a solid to liquid ratio of 1:10 (**a**) and 1:5 (**b**). Inset graph was the magnified profile of HMFCA production. Reaction conditions: 20 mL of phosphate buffer (200 mM, pH 6.0) containing 8 g_cdw_/L cells, *G. amansii* hydrolysates, and CaCO_3_ at half molar concentration of substrate, 35 °C and 200 rpm. Error bars represented standard deviations

Based on the obtained experimental results, the feasibility of co-production of D-galactonic acid and HMFCA from *G. amansii* hydrolysate was further investigated, which would contribute to the environmental protection due to the simple process for preparation of D-galactose and HMF without additional purification. Considering that the *G. amansii* hydrolysates might have an inhibitory effect on microbial cells, the bioconversion process was evaluated with different substrate concentrations. When *G. amansii* was pretreated under optimal conditions at a solid to liquid ratio of 1:10, a total of 41.24 g/L D-galactose and 7.85 g/L HMF were obtained. After mixed with wet cells of HMF-induced *P. putida* ATCC 47054, the initial concentration of D-galactose and HMF were diluted to 39.04 g/L and 7.29 g/L, respectively. Figure 5a depicted the change of D-galactonic acid and HMFCA yields along with reaction progress in the acid-pretreated hydrolysates of *G. amansii.* It revealed that the HMFCA yield increased very quickly to a maximum of 98.7% (corresponding to the overall yield of 39.2%) in 75 min, and the D-galactonic acid yield continuously rose until 7 h to a maximum of 95.2% (corresponding to the overall yield of 81.42%). We then improved the solid to liquid ratio to 1:5 in pretreatment process, in this case, 62.30 g/L D-galactose and 11.05 g/L HMF were produced. Similarly, the initial concentration of D-galactose and HMF were diluted to 56.36 g/L and 9.97 g/L individually after mixed with cells. Figure 5b showed that HMF was transformed completely in 105 min, with the maximum HMFCA yield of 98.7% (corresponding to the overall yield of 29.7%) and the highest HMFCA titer of 11.09 g/L. D-galactose was thoroughly bioconverted in 11 h, as the concentration of D-galactose was much higher than HMF in hydrolysates. The maximum D-galactonic acid yield was 91.1% (corresponding to the overall yield of 58.8%) and the highest D-galactonic acid titer was 55.30 g/L. The yields attained from *G. amansii* hydrolysates were slight lower than the results from commercial D-galactose and HMF.

In recent years, the exploration of macroalgae as a source for bio based chemicals and biofuels, including bioethanol [31], FDCA [32], levulinic acid [4], and hydrogen [7] etc., has attracted considerable interest. This study is the first report for D-galactonic acid and HMFCA production from macroalgae. The comparison of D-galactonic acid and HMFCA biosynthesis with previous reports was listed in Additional file 1: Table S2. Up to now, strains screened and applied for D-galactonic acid and HMFCA production are relatively few and mainly concentrated in some aerobic bacteria. Four different species of *Pseudomonas* were tested for their aldose-oxidizing activities and *P. fragi* TCCC 11892 was found to be an efficient producer of aldonic acids, including D-galactonic acid [14]. *G. oxydans* was another versatile species that can convert a series of biomass-derived sugars into corresponding aldonic acids [9, 33, 34]. But the enzymes responsible for D-galactose oxidation in *P. fragi* and *G. oxydans* were not unveiled. Liu et al. introduced D-galactose dehydrogenase from *P. syringae* and L-arabinose dehydrogenase from *Azospirillum brasilense* [29, 35], respectively, into *E. coli* to produce D-galactonate. To enhance the production of D-galactonate, inherent metabolic pathways for assimilating both D-galactose and D-galactonate must were blocked in advance [18, 29, 35]. With respect to HMFCA, Sayed et al. investigated and compared two *G. oxydans* strains, and one of them, *G. oxydans* DSM 50049, offered a much superior catalytic system for selective oxidation of HMF to HMFCA [26]. Zhang et al. isolated a new strain of *C. testosterone* SC1588 and proved its good biocatalytic oxidation of HMF to HMFCA [25]. They also spent much energy on identifying aldehyde dehydrogenases with ability to oxidize HMF [36]. *P. putida* ATCC 47054 was the first reported strain with selective biocatalytic activity towards the aldehyde group of both D-galactose and HMF, which were accomplished with utterly different enzymes. To sum up, taking advantage of the excellent efficiency and selectivity of *P. putida* ATCC 47054, efficient valorization of *G. amansii* biomass for dual production of D-galactonic acid and HMFCA at high yields was achieved for the first time, and these results further proved that algae biomass could be a promising feedstock for biochemicals production.

## Conclusions

The non-lignocellulosic feedstock of *G. amansii* was exploited for dual production of D-galactonic acid and HMFCA. *P. putida* ATCC 47054 exhibited high selectivity towards aldehyde oxidization of D-galactose and HMF, and its ability to biosynthesize HMFCA could be boosted by HMF. With *G. amansii* hydrolysates as substrate, a maximum of 55.30 g/L D-galactonic acid and 11.09 g/L HMFCA were co-produced with yields of 91.1% and 98.7%. This study provides a novel and potential implementation of *G. amansii* biomass for sustainable biochemicals production to supplement biorefinery of terrestrial feedstock.

## Materials and methods

### Compositional analysis of *G. amansii*

*G. amansii* was purchased from the local market of Nanjing, Jiangsu Province. The galactan, anhydrogalactan, glucan, acid-soluble lignin, and acid-insoluble lignin of *G. amansii* were 43.4%, 21.0%, 12.5%, 4.2%, and 1.0%, respectively, which was analyzed according to a modified National Renewable Energy Laboratory (Golden, CO, USA) standard procedure detailed in SI [5].

### Hydrolysis of *G. amansii*

*G. amansii* was washed with tap-water to remove salinity and impurities, air-dried, milled, and sieved between 20 and 80 mesh sizes. Afterwards, 2 g of *G. amansii* and 30 mL dilute hydrochloric acid solution with different concentration were mixed in a 100-mL hydrolysis vessel with screw cap at a solid to liquid ratio of 1:15 (w/v) at 120 °C for 1 h to investigate the feasibility of *G. amansii* hydrolysis. Effect of different pretreatment temperatures and times were tested. After pretreatment, the vessel was put into cool water immediately to terminate the reaction. Then, the mixture was centrifuged at 8000 *g* for 10 min and the aqueous phase was filtered (0.22 μm) before HPLC analysis.

### Whole cell catalyzed oxidation of D-galactose and HMF

*P. putida* ATCC 47054 was grown in LB medium at 30 °C with 200 rpm for 12 h. Then, 1% seed culture was inoculated to the fresh LB medium and cultivated under the same conditions. If HMF was used as inducer, 4.5 mM HMF was added when cell density was 1.5. After incubation for 12 h, the cells were harvested by centrifugation with 8000 *g* for 10 min and washed twice with phosphate buffer (200 mM, pH 6.0) prior to use in the oxidation reaction. The cell pellets were mixed with D-galactose or/and HMF to investigate their oxidation abilities. 5 mL of phosphate buffer (200 mM, pH 6.0) containing 8 g_cdw_/L cells, 50 g/L D-galactose or/and 10 g/L HMF was incubated at 35 °C and 200 rpm. In order to neutralize the acidic product, CaCO_3_ was added at the half molar concentration of substrate.

### Construct of *gcd*-disrupt mutant

The construction of a *gcd* disruption mutant was performed using a homologous recombination gene replacement system. All procedures were conducted according to a previous reference with minor modification [37]. Briefly, genomic DNA of *P. putida* ATCC 47054 was extracted through the Wizard Genomic DNA Purification Kit (Promega, Madison, WI, USA). The flanking regions of *gcd* gene were amplified from *P. putida* ATCC 47054 genomic DNA using the primers *gcd*up.f and *gcd*up.r (upstream, ~ 500 bp) and *gcd*down.f and *gcd*down.r (downstream, ~ 500 bp), respectively (Additional file 1: Table S3). After gel purification, overlap extension PCR was performed, wherein the upstream and downstream regions were fused using the primers *gcd*up.f and *gcd*down.r. The resulting PCR product was gel purified and ligated into the suicide vector pK18*mobsacB* to form a new plasmid pK18MS-Δ*gcd*. Plasmid pK18MS-Δ*gcd* was transformed into *P. putida* ATCC 47054 by electroporation. Single crossover recombinants containing the integration of plasmid pK18MS-Δ*gcd* into the chromosome of *P. putida* ATCC 47054 were obtained by plating the cells on LB agar containing 50 mg/L kanamycin. After two rounds of propagation in LB broth containing 15% (w/v) sucrose but without antibiotic, the double-crossover recombinants were screened by culture on LB agar containing 15% (w/v) sucrose. All the constructed strains were validated by PCR and DNA sequencing.

### Enzyme assay

*P. putida* ATCC 47054 and *gcd*-disrupt mutant were grown in LB medium at 30 °C and 200 rpm for 12 h on a rotary shaker. Then, 1% seed cultures were inoculated to the fresh LB medium and cultivated under the same conditions for 12 h. Thereafter, cells were harvested by centrifugation with 8000 *g* for 10 min and washed twice with 0.85% NaCl saline solution. The cell pellets were re-suspended in phosphate buffer (100 mM, pH 6.0) containing 1% (v/v) TritonX-100 and disrupted by sonication to obtain crude enzymes.

The activity of crude enzymes towards D-galactose was measured by monitoring the 3-(4,5-dimethylthiazol-2-yl)-2,5-diphenyltetrazolium bromide (MTT, ε = 8.8 per mM per cm) consumption at 590 nm [38, 39]. The reaction was performed in 1 mL of 100 mM phosphate buffer (pH 6.0), 100 mM D-galactose, 0.5 mM MTT, 0.5 mM phenazine ethosulfate, 1% (v/v) TritonX-100, and crude enzymes with suitable amount. One unit was defined as the amount of enzyme that reduced 1.0 μmol MTT per minute under the test condition. The concentrations of proteins were determined by the Bradford assay using bovine serum albumin as the standard.

### Whole cell catalyzed oxidation of *G. amansii* hydrolysates

*G. amansii* was pretreated under optimal conditions at a solid to liquid ratio of 1:5, and 1:10 (w/v). The hydrolysates were adjusted to pH 6.0 with NaOH prior to use as substrates. Bioconversions were performed in a larger volume using the above hydrolysates and the whole cells of *P. putida* ATCC 47054. 50 mL of phosphate buffer (200 mM, pH 6.0) containing 8 g_cdw_/L cells and hydrolysates of *G. amansii* was incubated at 35 °C and 200 rpm. CaCO_3_ was also added at half molar concentration of substrate.

### Analytical methods

The concentrations of d-glucose, HMF, HMFCA, levulinic acid, and formic acid in the reaction mixtures were determined by an HPLC system (Agilent 1260 series, USA) equipped with an Aminex HPX-87H column (Bio-Rad, USA) and a refractive index detector (Shimadzu, Japan) at 65 °C. The column was eluted with 5 mM H_2_SO_4_ at a flow rate of 0.6 mL/min and 55 °C. The concentrations of D-galactose and D-galactonic acid were analyzed on high performance anion-exchange chromatography (Dionex ICS-5000) linked to a CarboPac™ PA 200 column with NaOH and sodium acetate as eluents and a flow rate of 0.25 mL/min [9]. The AHG content was determined using the resorcinol-acetal method [40].

The yields of D-galactose and HMF were calculated as follows:

$${\text{D-Galactose yield }}\left( \% \right) \, = \frac{{{\text{D-Galactose produced }}\left( {\text{g}} \right)}}{{{\text{Original galactan (g) in G}} . {\text{amansii }}}} \times \frac{ 1 6 2}{ 1 8 0} \times 1 0 0$$

$${\text{HMF yield }}\left( \% \right) \, = \frac{{{\text{HMF produced }}\left( {\text{g}} \right)}}{{{\text{Original anhydrogalactan (g) in G}} . {\text{amansii }}}} \times \frac{ 1 4 4}{ 1 2 6} \times 1 0 0$$

The yields of D-galactonic acid and HMFCA were defined as the percentage of the measured product amount in the theoretical product amount based on the initial amount of commercial D-galactose and HMF or those in *G. amansii* hydrolysates, respectively. The overall yield of D-galactonic acid was calculated by multiplying D-galactose yield by D-galactonic acid yield, while the overall yield of HMFCA was calculated by multiplying HMF yield by HMFCA yield. All the experiments reported here were repeated independently at least twice. Both the mean values and corresponding standard deviations were presented.

## Supplementary information

**Additional file 1: Table S1.** Screening of five different *Pseudomonas* sp. strains for oxidation of D-galactose and HMF. **Table S2.** Comparison of D-galactonic acid and HMFCA biosynthesis from different substrates. **Table S3.** Plasmids and oligonucleotide primers for *gcd* disruption.

## Data Availability

All data generated or analyzed during this study are included in this published article.

## Abbreviations

HMF: 5-hydroxymethylfurfural
HMFCA: 5-hydroxymethyl-2-furancarboxylic acid
FDCA: 2,5-furandicarboxylic acid
PQQ-GCD: Pyrroloquinoline quinone-dependent glucose dehydrogenase
AHG: 3,6-anhydro-l-galactose
