# Supplementary material for: Valorization of Gelidium amansii for dual production of D-galactonic acid and 5-hydroxymethyl-2-furancarboxylic acid by chemo-biological approach
Source: Microb Cell Fact. 2020 May 14;19:104. doi: 10.1186/s12934-020-01357-6 (PMC7227364; doi:10.1186/s12934-020-01357-6)
Supplement: Supplementary file 1 — Additional file 1: Table S1. Screening of five different Pseudomonas sp. strains for oxidation of D-galactose and HMF. Table S2. Comparison of D-galactonic acid and HMFCA biosynthesis from different substrates. Table S3. Plasmids and oligonucleotide primers for gcd disruption. [file 12934_2020_1357_MOESM1_ESM.docx]

**Additional file for**

**Valorization of *Gelidium amansii* for dual production of D-galactonic acid and 5-hydroxymethyl-2-furancarboxylic acid by chemo-biological approach**

Peng Liu^1^, Jiaxiao Xie^1^, Huanghong Tan^3^, Feng Zhou^1^, Lihua Zou^3^, Jia Ouyang^2,3,4*^

^1^*College of Forestry, Nanjing Forestry University, Nanjing 210037, People’s Republic of China*

^2^*Key Laboratory of Forestry Genetics & Biotechnology (Nanjing Forestry University), Ministry of Education, Nanjing 210037, People’s Republic of China*

^3^*Jiangsu Co-Innovation Center of Efficient Processing and Utilization of Forest Resources, College of Chemical Engineering, Nanjing Forestry University, Nanjing 210037, People’s Republic of China*

^4^*Jiangsu Province Key Laboratory of Green Biomass-based Fuels and Chemicals, Nanjing 210037, People’s Republic of China*

^*^Corresponding author. Address: College of Chemical Engineering, Nanjing Forestry University, Nanjing 210037, People’s Republic of China, Tel.: 86-025-85427129, Fax: 86-025-85427587, E-mail: hgouyj@njfu.edu.cn.

**Materials**

*G. amansii* was purchased from the local market of Nanjing, China. HMF and HMFCA were purchased from Adamas Reagent Ltd (Shanghai, China). All chemicals were of analytical grade. The bacterial strains, *P. putida* ATCC 47054 and *P. fragi* ATCC 4973 were obtained from American Type Culture Collection; *P. stutzeri* CICC 10402 and *P. rhodesiae* CICC 21960 were obtained from China Center of Industrial Culture Collection; *P. aeruginosa* CGMCC 1.10712 was obtained from China General Microbiological Culture Collection Center.

**Compositional analysis of *G. amansii***

The composition of *G. amansii* includes cellulose, agarose, lignin, and others. They were analyzed according to a modified NREL protocol [1]. First, *G. amansi* samples were hydrolyzed using 72% sulfuric acid at 30°C for 30 min, followed by 4% sulfuric acid at 121°C for an additional 1 h. After two-stage acid hydrolysis, the concentrations of D-glucose and D-galactose in the liquid phase were analyzed by HPLC. Concerning lignin, the acid-insoluble lignin fraction was gravimetrically determined by the mass of the solid residues remaining after acid hydrolysis; while the acid-soluble lignin was determined by UV absorption of liquid phase.

The AHG content was determined using a modified resorcinol-acetal method with fructose as standard [2]. First, the oven-dried samples of 0.4 mg were thoroughly mixed with 20 mL resorcinol-acetal reagent in screw-capped test tubes, and placed on an ice bath for at least 3 min. The tubes were removed for 4 min at 20°C and then submerged in hot water at 80°C for 10 min. Finally, the mixtures were cooled on ice for 1.5 min and the absorbance at 555 nm was recorded within 15 min. A reference standard curve was constructed using fructose.

**Comparison of different strains for oxidation of D-galactose and HMF**

The cultivation method for *P. stutzeri* CICC 10402, *P. aeruginosa* CGMCC 1.10712, *P. rhodesiae* CICC 21960 and *P. fragi* ATCC 4973 was same as *P. putida* ATCC 47054. For biocatalyst preparation, all these strains were cultured with 200 rpm for 12 h on a rotary shaker. Then, 1% seed culture was inoculated to the fresh LB medium and cultivated under the same conditions. After incubation for 12 h, the cells were harvested by centrifugation with 8,000 *g* for 10 min and washed twice with phosphate buffer (200 mM, pH 6.0) prior to use in the oxidation reaction. The cell pellets of different *Pseudomonas* sp. strains collected were mixed with D-galactose or HMF to investigate their oxidation abilities. 5 mL of phosphate buffer (200 mM, pH 6.0) containing 8 g_cdw_/L cells, 50 g/L D-galactose or 10 g/L HMF was incubated at 35°C and 200 rpm. In order to neutralize the acidic product, CaCO_3_ was added at the half molar concentration of substrate. Aliquots were withdrawn from the reaction mixtures at specified times and diluted with the corresponding mobile phase prior to HPLC analysis.

Table S1 Screening of five different *Pseudomonas* sp. strains for oxidation of D-galactose and HMF.

|  | D-Galactonic acid | HMFCA |
| --- | --- | --- |
| *P. putida* ATCC 47054 | +++ | +++ |
| *P. fragi* ATCC 4973 | +++ | – |
| *P. stutzeri* CICC 10402 | + | – |
| *P. rhodesiae* CICC 21960 | +++ | ++ |
| *P. aeruginosa* CGMCC 1.10712 | +++ | – |

Symbol '+' represents the strains possess the ability to oxidize D-galactose or HMF, while symbol '–' represents they lack such ability. More '+' represents stronger ability. Reaction conditions: 5 mL of phosphate buffer (200 mM, pH 6.0) containing 8 g_cdw_/L cells, 50 g/L D-galactose or 10 g/L HMF, 35°C and 200 rpm.

Table S2 Comparison of D-galactonic acid and HMFCA biosynthesis from different substrates.

| Substrate | Biocatalyst | D-Galactonic acid (g/L) | HMFCA (g/L) | Reference |
| --- | --- | --- | --- | --- |
| D-Galactose | *G. oxydans* NL71 | 62.2 (48 h) | – | 3 |
| D-Galactose from cheese whey power hydrolysates | *G. oxydans* NL71 | 84.2 (44 h) | – | 3 |
| D-Galactose | *E. coli* harboring D-galactose dehydrogenase | 17.6 (72 h) | – | 4 |
| D-Galactose | *E. coli* harboring L-arabinose dehydrogenase | 24.0 (72 h) | – | 5 |
| HMF | *C. testosterone* SC1588 | – | 22.3 (36 h) | 6 |
| Home-made HMF from fructose | *G. oxydans* DSM 50049 | – | 44.6 (23 h) | 7 |
| HMF | *Deinococcus wulumuqiensis* R12 | – | 38.3 (36 h) | 8 |
| Home-made HMF from fructose | *Serratia marcescens* | – | 0.79 (20 h) | 9 |
| HMF | *E. coli* harboring HMF oxidase mutant | – | 20.7 (96) | 10 |
| HMF | *E. coli* harboring vanillin dehydrogenase | – | 26.1 (12 h) | 11 |
| *G. amansii* acid-hydrolysates | *P. putida* ATCC 47054 | 55.30 (11 h) | 11.09 (105 min) | This study |

Table S3 Plasmids and oligonucleotide primers for *gcd* disruption.

| Name | Relevant characteristic |
| --- | --- |
| Plasmids |  |
| pK18*mobsacB* | Allelic exchange vector, *ori*ColE1 Mob^+^, *lacZα*, *sacB*; Km^r^ |
| pK18MS-Δ*gcd* | The flanking regions of *gcd* gene were inserted into pK18*mobsacB* |
| Primers |  |
| *gcd*up.f | GAATTCATGAGCACTGAAGGTGCGAACCAAGG (*EcoR* I) |
| *gcd*up.r | CAACCTGCTCACGCGGGACCATCTTGCCCCGGCCATG  CCCGACGGCGAAT |
| *gcd*down.f | ATTCGCCGTCGGGCATGGCCGGGGCAAGATGGTCCCG  CGTGAGCAGGTTG |
| *gcd*down.r | GGATCCTTACTCGGCTAATTTGTAAGCAATG (*BamH* I) |

**Reference**

1. Weldemhret TG, Nisola GM, Valdehuesa KNG, Lee WK, Ramos KRM, Chung WJ. Ionic liquid pre-treatment in tandem with recombinant agarase cocktail saccharification of *Gelidium amansii* for D-galactose and 3,6-anhydro-L-galactose production. ACS Sustain Chem Eng. 2019;7:7563-7571.
2. Yaphe W, Arsenault GP. Improved resorcinol reagent for the determination of fructose, and of 3,6-anhydrogalactose in polysaccharides. Anal Chem. 1965;13:143-148.
3. Zhou X, Hua X, Huang L, Xu Y. Bio-utilization of cheese manufacturing wastes (cheese whey powder) for bioethanol and specific product (galactonic acid) production via a two-step bioprocess. Bioresour Technol. 2019;272:70-76.
4. Liu H, Ramos KRM, Valdehuesa KNG, Nisola GM, Malihan LB, Lee WK, Park SJ, Chung WJ. Metabolic engineering of *Escherichia coli* for biosynthesis of D-galactonate. Bioproc Biosyst Eng. 2014;37:383-391.
5. Liu H, Valdehuesa KNG, Ramos KRM, Nisola GM, Lee WK, Chung WJ. L-arabonate and D-galactonate production by expressing a versatile sugar dehydrogenase in metabolically engineered *Escherichia coli*. Bioresour Technol. 2014;159:455-459.
6. Zhang XY, Zong MH, Li N. Whole-cell biocatalytic selective oxidation of 5-hydroxymethylfurfural to 5-hydroxymethyl-2-furancarboxylic acid. Green Chem. 2017;19:4544-4551.
7. Sayed M, Pyo SH, Rehnberg N, Hatti-Kaul R. Selective oxidation of 5-hydroxymethylfurfural to 5-hydroxymethyl-2-furancarboxylic acid using *Gluconobacter oxydans*. ACS Sustain Chem Eng. 2019;7:4406-4413.
8. Cang R, Shen LQ, Yang G, Zhang ZD, Huang H, Zhang ZG. Highly selective oxidation of 5-hydroxymethylfurfural to 5-hydroxymethyl-2-furancarboxylic acid by a robust whole-cell biocatalyst. Catalysts. 2019;9:526.
9. Muñoz T, Rache LY, Rojas HA, Romanelli GP, Martinez JJ, Luque R. Production of 5-hydroxymethyl-2-furan carboxylic acid by *Serratia marcescens* from crude 5-hydroxymethylfurfural. Biochem Eng J. 2020;154:107421.
10. Wang ZW, Gong CJ, He YC. Improved biosynthesis of 5-hydroxymethyl-2-furancarboxylic acid and furoic acid from biomass-derived furans with high substrate tolerance of recombinant *Escherichia coli* HMFOMUT whole-cells. Bioresour Technol. 2020;303:122930.
11. Zhang XY, Ou XY, Fu YJ, Zong MH, Li N. Efficient synthesis of 5-hydroxymethyl-2-furancarboxylic acid by *Escherichia coli* overexpressing aldehyde dehydrogenases. J Biotechnol. 2020;307:125-130.
